# Mild internet use is associated with epigenetic alterations of key neurotransmission genes in salivary DNA of young university students

**DOI:** 10.1038/s41598-023-49492-5

**Published:** 2023-12-14

**Authors:** Eugenia Annunzi, Loreta Cannito, Fabio Bellia, Francesca Mercante, Matteo Vismara, Beatrice Benatti, Alberto Di Domenico, Riccardo Palumbo, Walter Adriani, Bernardo Dell’Osso, Claudio D’Addario

**Affiliations:** 1 Department of Neuroscience, Imaging and Clinical Sciences, University “G. d’Annunzio” of Chieti-Pescara, 66100 Chieti, Italy; 2 Department of Humanities, University of Foggia, Foggia, Italy; 3 Center for Advanced Studies and Technology, University “G. d’Annunzio” of Chieti-Pescara, 66100 Chieti, Italy; 4 Department of Bioscience and Technology for Food, Agriculture and Environment, University of Teramo, 64100 Teramo, Italy; 5 Department of Biological Sciences, Fordham University, Bronx, NY USA; 6 Department of Psychiatry, Department of Biomedical and Clinical Sciences “Luigi Sacco”, University of Milan, ASST Fatebenefratelli-Sacco, 20019 Milan, Italy; 7 “Aldo Ravelli” Center for Nanotechnology and Neurostimulation, University of Milan, Milan, Italy; 8 Department of Psychological, Health and Territorial Sciences, University “G. d’Annunzio” of Chieti-Pescara, 66100 Chieti, Italy; 9 Center for Behavioural Sciences and Mental Health, Istituto Superiore di Sanità, Viale Regina Elena, 299, 00161 Rome, Italy; 10 Department of Clinical Neuroscience, Karolinska Institute, 10316 Stockholm, Sweden

**Keywords:** Epigenetics, Predictive markers, Methylation analysis, Epigenetics and behaviour

## Abstract

The potentially problematic use of the Internet is a growing concern worldwide, which causes and consequences are not completely understood yet. The neurobiology of Internet addiction (IA) has attracted much attention in scientific research, which is now focusing on identifying measurable biological markers. Aim of this study was to investigate epigenetic and genetic regulation of oxytocin receptor (*OXTR*), dopamine transporter (*DAT1*) and serotonin transporter (*SERT*) genes using DNA obtained from saliva samples of young university students: the Internet Addiction Test (IAT) was administered to evaluate the potential existence and intensity of IA. Significant changes in DNA methylation levels at *OXTR*, *DAT1* and *SERT* genes were observed in the 30 < IAT < 49 group (mild-risk internet users) compared to the IAT < 29 subjects (complete control of internet use) and IAT > 50 subjects (considered as moderately addicted). Moreover, epigenetic markers were significantly correlated, either directly (for *OXTR* and *DAT1*) or inversely (*OXTR* and *DAT1* versus *SERT*), to the psychometric properties. Our data confirmed the association of *OXTR*, *DAT1* and *SERT* genes in processes related to behavioural addictions and might be of relevance to suggest possible biological predictors of altered behaviours and the eventual vulnerability to develop an IA. Different other genetic pathways have been suggested to play a role in IA and research is ongoing to better define them, in order to help in the early diagnosis as well as in the development of new potential treatments.

## Introduction

Excessive Internet use, problematic or truly addictive, is a growing concern worldwide^1,2^ and researchers are focusing the attention on the development and progression of Internet Addiction (IA) which causes and consequences are not yet completely understood. IA is defined as an impulse-control disorder that does not involve an intoxicant^3^ and it has been associated with pathological conditions like attention deficit hyperactivity disorder, depression and anxiety^4–7^. In general, Internet usage monitoring is of particular relevance in adolescents and young adults, major users of this technology. Moreover, considering developmental changes occurring in their brain, especially in cognition, stress, and motivations, young adults are more vulnerable to addictive behaviours^8^ and, ultimately, this might determine poor concentration and impact academic performance^9^.

Research is nowadays focusing on the understanding of the neurobiology of IA and on the identification of measurable biological markers that might help in the early diagnosis as well as in the development of new potential treatments^10^. IA shares neurobiological mechanisms with other addictive behaviours^11^. For instance, excessive Internet use has been associated with an overall deficiency in the reward system, related to a reduced dopaminergic activity^12^. A previous study showed an altered resting-state glucose metabolism in dopamine projecting brain regions in subjects with Internet game overuse^13^.

An important role in dopaminergic system regulation is played by the dopamine transporter (DAT), situated in the presynaptic terminal and responsible for the active dopamine (DA) reuptake^14^. Considering addictive behaviours, the expression of *DAT1* (the gene encoding DAT) resulted to be reduced in the striatum of individuals with IA compared to controls^15^. The epigenetic regulation of *DAT1* gene promoter region was also investigated in relation to excessive use of the Internet in school-age youths and pre-adolescents^16^ as well as in youths (from 18 to 34 years)^17^. Of note, the human *DAT1* gene is also characterized by a polymorphic 40-base pair (bp) variable number of tandem repeats (VNTR) in the 3’-untranslated region (3’-UTR), previously associated with impulsivity and drug dependence/addiction^18,19^.

Serotonin (5-hydroxytryptamine [5HT]) system modulation has also been studied in relation to IA. In particular, the serotonin reuptake transporter gene (*SERT*), also harbouring in the promoter region a VNTR (5-HTTLPR)^20^, with the homozygous long allelic variant (L) associated with higher *SERT* mRNA levels and greater rate of reuptake compared to the short allelic variant (S)^20,21^. Of relevance in the frame of this work, Lee et al.^22^ reported that the S allele is more frequently expressed in excessive Internet users. More recently, *SERT* levels were reported to be higher in students reporting stress and with an IA diagnosis compared to stressed students but not IA^23^.

We also focused the attention on the oxytocinergic system, whose role in the regulation of social interactions, occurring also online, and empathy in adolescents and young adults has been established^24^. Moreover, according to the Affective Neuroscience Theory framework, oxytocin (*OXT*) was suggested as a promising candidate in IA^25^ and *OXT* secretion resulted increased in the blood of adolescents with Internet gaming disorder^26^. The *OXTR* gene contains 4 exons, with the last 2 encoding for the receptor^27^. Remarkably, *OXTR* DNA methylation at exon 3 has been suggested to be responsive to environmental stimuli^28–30^.

Thus, several pieces of evidence show that epigenetic mechanisms describe gene-environment interactions leading to the development of different phenotypes^31^, including susceptibility to IA^32^.

The Internet Addiction Test (IAT) has been the first validated and the most widely used instrument to measure symptoms and severity of IA^3^ developed as a unidimensional scale. However, since the number of extracted factors and factor arrangements varied across studies, a multidimensional nature of the IA construct was suggested by Fioravanti and colleagues^33^. Analysing students aged between 14 and 26 years old, good psychometric properties of the IAT have been observed and yielded to two-factors: the first factor “Emotional and cognitive preoccupations with the Internet and social consequences” is related to the emotional and cognitive salience with the negative social consequences due to Internet use, and the second factor “Loss of control and interference with daily duties” is related to unsuccessful attempts to control the amount of time spent online and to the negative consequences of the Internet use on daily functioning^33^. We decided to consider in our study sample also the dimensionality of the Italian version of the IAT.

In the present work we investigated in young university students showing different scores in IAT test the epigenetic regulation of *OXTR*, *DAT1* and *SERT* genes, and for these two latter also the role of genetic analysing the two VNTRs mentioned-above, using DNA obtained from saliva samples. It is important to point out that saliva samples, which collection is clearly less invasive and without the potential risks occurring when using blood^34,35^, are now considered a valuable alternative to blood for molecular assays^36^, also considering the high DNA quality required for genetic and epigenetic analysis^37–41^. The aim of this study was thus to define possible biological factors predisposing to the development of IA focusing on the genetic and epigenetic regulation of key genes. Specifically, we attempt to understand if genetic variants, assumed to be irreversible, and/or epigenetic marks, potentially reversible, might be used as tools to monitor the development and/or the progression of IA.

## Material and methods

### Participants

A total sample of 84 volunteers (Males = 20; Females = 64; Mean age: 20.1 ± 2.09 years) were recruited through a public announcement across different majors in University of Chieti. During the recruitment phase, students who expressed interest in participating were sent a short preliminary questionnaire regarding exclusion criteria, which included: (i) having previously received a diagnosis for a psychiatric disorder; (ii) previously or currently taking medication for a psychiatric disorder; (iii) having previously or currently taken drugs or psychotropic substances.

This information was obtained by means of a direct question. All participants who answered yes to one or more of these questions were preliminarily excluded from the study.

Students provided written informed consent and received no monetary or credit compensation for their participation.

### Procedure

The research complies with the Declaration of Helsinki and the research protocol was approved by the Institutional Review Board of Psychology (IRBP) of the Department of Psychological, Health and Territorial Sciences at G. d’Annunzio University of Chieti-Pescara (identification code: 20,026; date of approval: 19 February 2021). After providing their informed consent, participants were invited to the lab for the saliva collection procedure (see salivary sample collection paragraph for more details) and were provided an alphanumeric univocal code to be used in the subsequent online survey completion. The online survey was administered through an online platform (Qualtrics, Provo, UT) and included demographic questions (gender, age) and the Internet Addiction Test (IAT). To address the issue of possible multiple completion from the same participant, together with alphanumeric code check, completion of the survey from the same IP address was prevented. Also, to ensure that participants keep focused on response during the survey completion, a control question (asking the participants to flag a specific response option) was added. Based on this control question, no participants were excluded. The students who did not complete the survey (N = 2) were instead excluded from the study and from following analysis, thus obtaining a total sample of 84 participants.

### Internet addiction test

The Internet Addiction Test (IAT)^3^ is the most used instrument to investigate and assess the severity of IA both in clinical practice and research. The IAT is a 20-item 5-point Likert scale 5 (0 = not applicable, 5 = always) covering Internet use habits, preoccupation with its use, ability to control online use, and the extent of lying or hiding about online use and related problems in everyday functioning. Total IA scores are calculated with a maximum score of 100, according to the Italian validation^42^. Students were asked to consider only the time spent online for non-academic purposes. IAT score from 0 to 29 represent average users with complete control of their internet use; scores from 30 to 49 represent the presence of a mild level of internet use that might fall into addiction; subjects which score results above 50 are considered addicted: moderately (from 50 to 79) or severely (from 80 to 100). The scale reliability in this study showed very good internal consistency, obtaining a Cronbach’s alpha of 0.82.

### Psychometric factors of the Italian IAT

The items of the IAT Italian version were divided in two factors^33^. The first factor (F1), ‘‘Emotional and cognitive preoccupations with the Internet and social consequences” encompasses items related to the emotional and cognitive salience of Internet use, and items concerned with the negative social consequences due to Internet use (item 3, 9, 10, 11, 12, 13, 15, 17, 18, 19, 20). The second factor (F2), ‘‘Loss of control and interference with daily duties,’’ contains items related to unsuccessful attempts to control the amount of time spent online and to the negative consequences of the Internet use on daily functioning (item 1, 2, 4, 5, 6, 7, 8, 14, 16).

### Molecular studies

#### Salivary samples collection

Before completing the survey, participants’ self-collected saliva samples by using a 15 ml centrifuge tube where the subject spit roughly 2 ml of saliva. Saliva was chosen since it has technical advantages over blood, particularly for its non-invasive sampling method and several molecular measures in saliva might reflect those in blood^35^. To ensure a correct sampling, during the recruiting phase participants were asked not to take food, drugs, drinks (besides water), or use lip products as well as not to smoke or brush their teeth at least two hours before arriving at the laboratory to avoid possible contamination. Each sample was then stored at − 80 °C before being analysed.

### DNA extraction and methylation study

Genomic DNA was extracted using the salting-out method^43^ and the NanoDrop 2000c UV–Vis Spectrophotometer (ThermoFischer Scientific, Waltham, MA, USA) was used to assess the quantity and quality of each sample. Each purified DNA was subjected to bisulfite modification by means of the EZ DNA Methylation-GoldTM Kit (Zymo Research, Orange, CA, USA), and the DNA methylation status of each of the CpG sites was assessed using a pyrosequencing assay. Bisulfite-treated DNA was first amplified by the PyroMark PCR Kit (Qiagen, Hilden, Germany) with a biotin labeled primer (see Table 1) according to the manufacturer’s recommendations. PCR conditions were as follows: 95 °C for 15 min, followed by 45 cycles of 94 °C for 30 s, 56 °C for 30 s, 72 °C for 30 s, and, finally, 72 °C for 10 min. Specificity of PCR products was then verified by electrophoresis. The sequencing was performed on a PyroMark Q48 Autoprep using Pyro Mark Gold reagents (Qiagen, Hilden, Germany). The methylation’s level was analysed through the PyroMark Q48 Autoprep version 2.4.2 software which calculates the methylation percentage mC/(mC + C) (mC = methylated cytosine, C = unmethylated cytosine) for each CpG site, allowing quantitative comparisons. The primers used are reported in the table below.

**Table 1:** Sequences considered for DNA methylation analysis. GeneGlobe Identification numbers refer to the assay designed for the analysis. Detailed genomic locations are reported.

	GeneGlobe ID	Sequence analysed	CpG sites	Genomic location
*OXTR*	PM00016821	AGGCGGCACAGCAGGTCGGGCCCGTAGAAGCGGA	4	Chr3, bp 8,767,880–8,767,847
*DAT1*	PM00022064	GCGGCGGCGGCTTGCCRGAGACTCGCGAGCTCCGC	6	Chr5, bp 1,444,718–1,444,679
*SERT*	PM00065625	CCCCGACACACACACACGCTCGCAGGGAGGAGCGGAGCGCGGA	6	Chr17, bp 30,235,935–30,235,893

### 5-HTTLPR and VNTR study

For genotyping 5-HTTLPR, the PCR was performed in a final volume of 10 µl containing 5 µl Master Mix 2X (Promega, UK), 0.5 µl 10 mM 5-HTTLPR forward primer, 0.5 µl 10 mM 5-HTTLPR reverse primer, 2 µl H2O nuclease-free and 2 µl of DNA samples. The following primers for PCR were used: 5-HTTLPR fwd: 5′-CGTTGCCGCTCTGAATGC-3′, 5-HTTLPR rev: 5′-TGGTAGGGTGCAAGGAGAATG-3′^44^. Cycling conditions were as follows: 95 °C for 15 min, followed by 45 cycles of 94 °C for 30 s, 56 °C for 30 s, 72 °C for 30 s, and, finally, 72 °C for 10 min. For the detection of PCR fragments, gel electrophoresis on 2% agarose gel stained with GelRed® Nucleic Acid Gel Stain—Biotium was used and then visualized using the Gel Doc Systems from Bio-Rad. We detected PCR products of 340 bp for the Short allele and band sizes of 384 bp for the Long allele.

The detection of 40-bp VNTR in the 3′-untranslated region of the *DAT1* gene was performed using the method described by Shinohara et al. (2004). The PCR was performed in 10 µl reactions as described above and the following primers were used: VNTR *DAT1* fwd: 5′-TGTGGTGTAGGGAACGGCCTGAG-3′, VNTR *DAT1* rev: 5′-CTTCCTGGAGGTCACGGCTCAAGG-3′^45^. The detection of PCR fragments was performed as for the 5-HTTLPR. We detected the 360 bp fragment (7 repeat allele), 400 bp fragment (8 repeat allele), 440 bp fragment (9 repeat allele), and 480 bp fragment (10 repeat allele).

### Statistical analysis

The data are expressed as mean ± standard error of the mean (SEM). The One-Way ANOVA test was used for DNA methylation statistical analysis. To reduce type I error and avoid false positives, a Bonferroni correction was applied to determine the confidence intervals when making pairwise comparison of the means for each data set. Hardy Weinberg equilibrium for genotype analysis was assessed using the Chi-Square test. Correlation analysis between DNA methylation levels and IAT or psychometric factors was performed with Spearman’s correlation coefficient. The *p*-values < 0.05 were considered statistically significant. A power analysis on an R package, WebPower^46^, was performed, and the results suggested a good power for each path (≥ 0.8).

## Results

### Sample characteristics

On the basis of the IAT cut-offs defined by Young^3^, the total sample (N = 84) was divided into 3 groups based on IA severity: a group with no problems using the Internet (IAT < 29) (N = 17), a group with mild severity of IA (30 < IAT < 49) (N = 50) and a third group with moderate severity of IA (50 < IAT < 80) (N = 17). Among the students who participated in the study, we found no subjects with IAT scores higher than 80. Considering the gender of the participants, we observed that there was a similar woman to men ratio in the three groups: IAT < 29: 13 women (72.22%) and 5 men (27.78%); 30 < IAT < 49: 40 women (80%) and 10 men (20%); IAT > 50: 11 women (68.75%) and 5 men (31.25%). Also age was quite similar in the three groups: IAT < 29: 20.78 ± 0.58; 30 < IAT < 49: 19.68 ± 0.25; IAT > 50: 20.88 ± 0.61, (average year ± SD).

### DNA methylation studies

**Figure 1:**
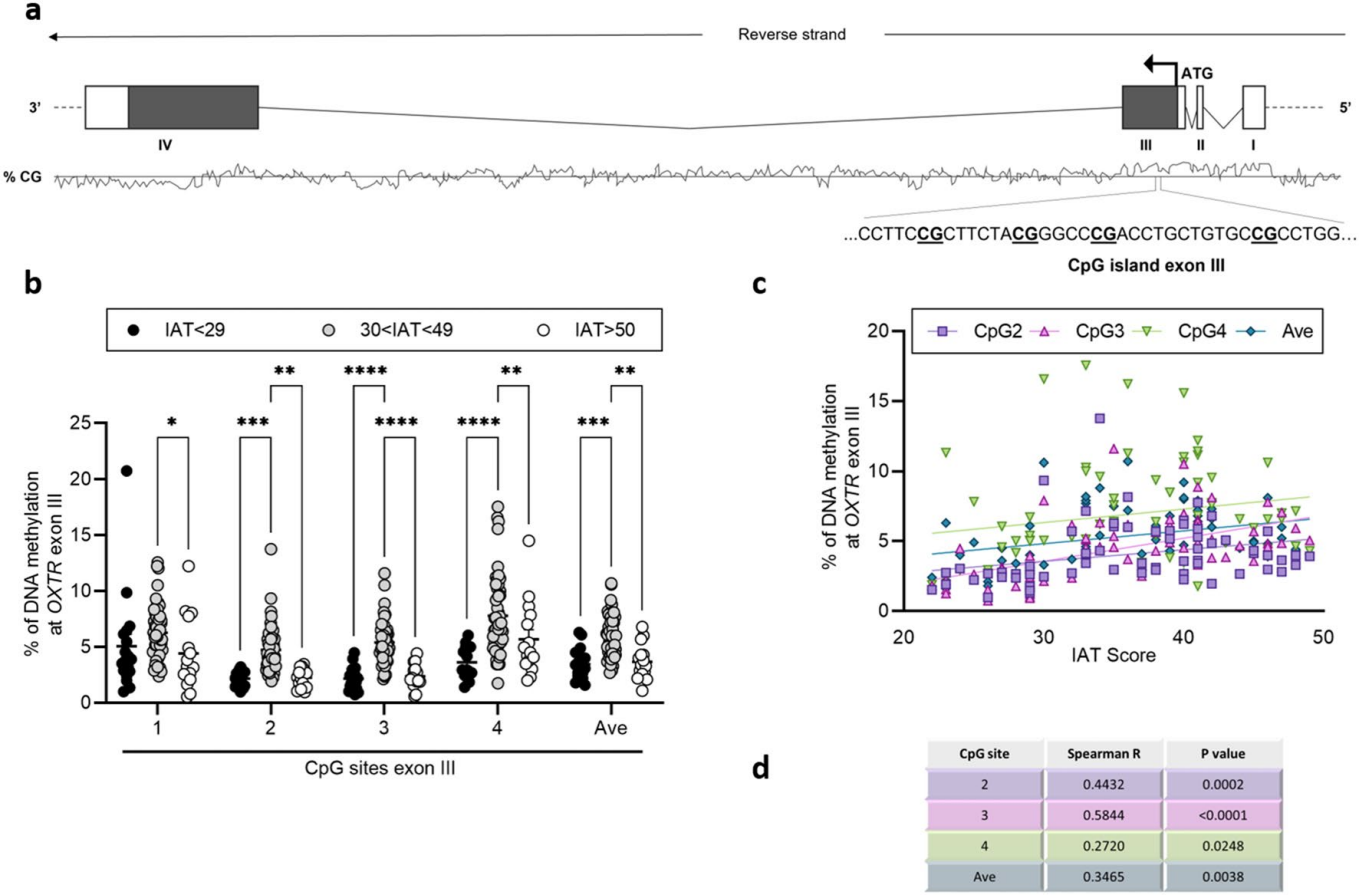
(**a**) Schematic representation of the human *OXTR* gene. Boxes represent the exons, with the translated part, beginning on the ATG site, filled in dark grey. In the lower part is reported the CpG island located at exon III in which are highlighted the 4 CpG sites under study. (**b**) DNA methylation levels in *OXTR* exon III in saliva samples of young adults with IAT < 29, 30 < IAT < 49 and IAT > 50, represented as scattered dot plots (mean ± SEM, of each group) for the individual CpG sites and the average (Ave) of the 4 CpG sites included in the study. Significant differences are indicated (Bonferroni corrected *p < 0.05, **p < 0.01, ***p < 0.005, ****p < 0.001). (**c**) Correlation between % of DNA methylation at *OXTR* exon III (Y bar) and IAT score (X bar). Data were compared by Spearman’s rank correlation coefficient; Spearman’s r and *p* values for each CpG site are reported in (**d**).

We report specific and significant alterations in the % of *OXTR*, *DAT1* and *SERT* DNA methylation at different CpG sites. Considering *OXTR* gene (schematic representation Fig. 1a), an increase in DNA methylation was observed at CpG 2 (30 < IAT < 49: 4.73 ± 0.29; IAT < 29: 2.18 ± 0.15; *p* = 0.0004), at CpG 3 (30 < IAT < 49: 5.41 ± 0.26; IAT < 29: 2.17 ± 0.24; *p* < 0.0001), at CpG 4 (30 < IAT < 49: 7.80 ± 0.50; IAT < 29: 3.62 ± 0.38; *p* < 0.0001), and at the average of the 4 CpG sites (30 < IAT < 49: 6.06 ± 0.26; IAT < 29: 3.46 ± 0.32; *p* = 0.0003) analysed (Fig. 1b), in young adults with IAT score ranging between 30 and 49 when compared to those with IAT < 29. Furthermore, we observed an increase in DNA methylation for all the CpG sites in the 30 < IAT < 49 group when compared to IAT > 50: CpG1 (30 < IAT < 49: 6.29 ± 0.35; IAT > 50: 4.42 ± 0.85; *p* = 0.025), CpG2 (30 < IAT < 49: 4.73 ± 0.29; IAT > 50: 2.25 ± 0.22; *p* = 0.0015), CpG3 (30 < IAT < 49: 5.41 ± 0.27; IAT > 50: 2.39 ± 0.27; *p* < 0.0001), CpG4 (30 < IAT < 49: 7.80 ± 0.51; IAT > 50: 5.69 ± 0.86; *p* = 0.009), average (30 < IAT < 49: 6.06 ± 0.27; IAT > 50: 3.69 ± 0.40; *p* = 0.0025).

**Figure 2:**
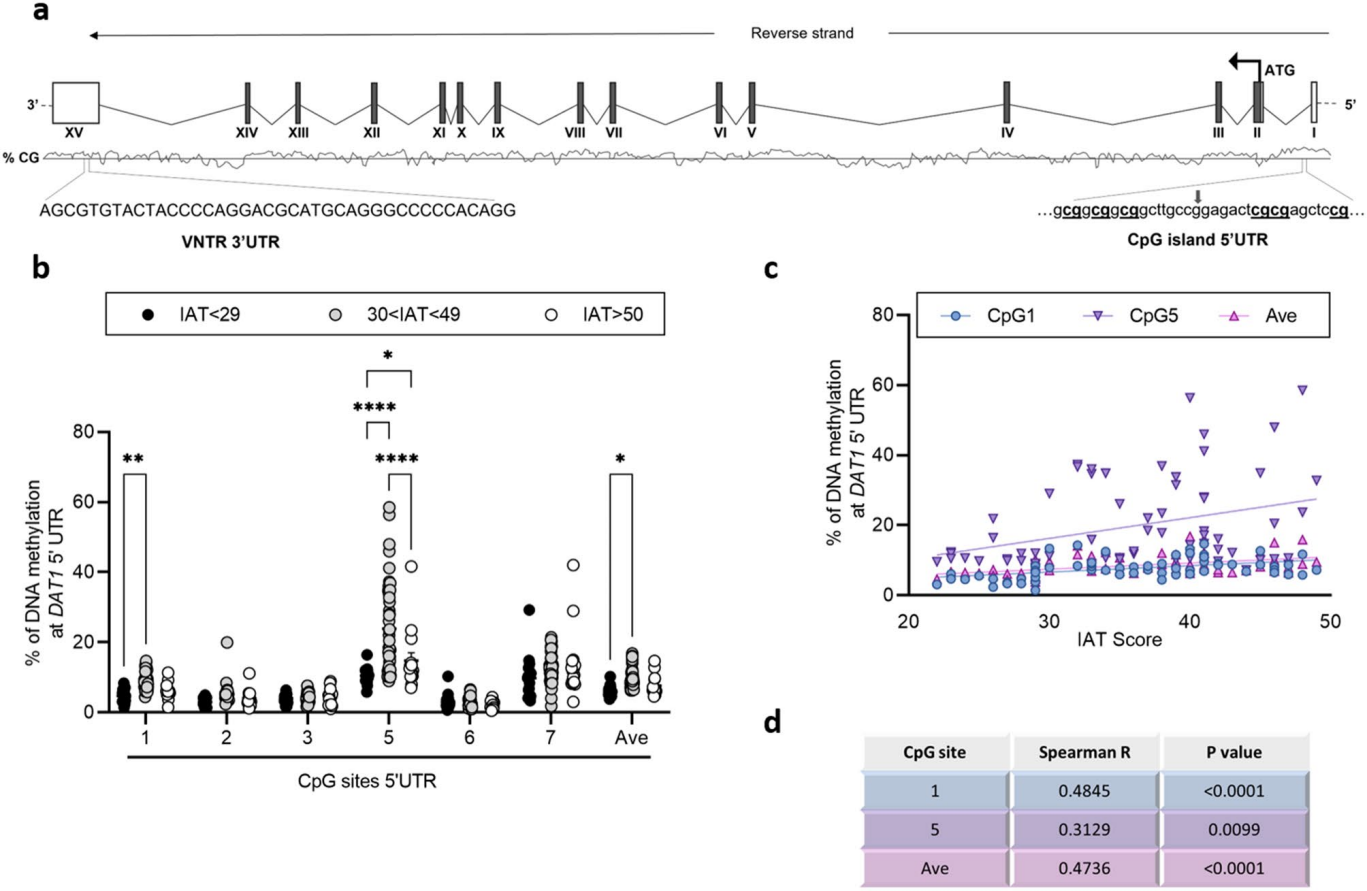
(**a**) Schematic representation of the human *DAT1* gene. Boxes represent the exons, with the translated part, beginning on the ATG site, filled in dark grey. In the lower part, on the right is reported the CpG island located into the 5′UTR in which are highlighted the 6 CpG sites under study with the arrow indicating the rs2911493 and on the left is reported the region containing the VNTR analysed in the subjects. (**b**) DNA methylation levels in *DAT1* 5’UTR region in saliva samples of young adults with IAT < 29, 30 < IAT < 49 and IAT > 50, represented as scattered dot plots (mean ± SEM, of each group) for the individual CpG sites and the average (Ave) of the 6 CpG sites included in the study; CpG site 4 was not included in the study for the presence of the rs2911493). Significant differences are indicated (Bonferroni corrected *p < 0.05, **p < 0.01, ****p < 0.001). (**c**) Correlation between % of DNA methylation at *DAT1* 5’UTR (Y bar) and IAT score (X bar). Data were compared by Spearman’s rank correlation coefficient; Spearman’s r and *p* values for each CpG site are reported in (**d**).

Regarding *DAT1* gene (schematic representation Fig. 2a), a significant increase of DNA methylation levels in students with intermediate IAT score compared to the IAT < 29 was observed at CpG 1 (30 < IAT < 49: 8.79 ± 0.35; IAT < 29: 4.71 ± 0.41; *p* = 0.007), CpG 5 (30 < IAT < 49: 23.87 ± 1.95; IAT < 29: 10.50 ± 0.56; *p* < 0.0001) and at the average of the 6 CpG sites (30 < IAT < 49: 9.42 ± 0.39; IAT < 29: 5.93 ± 0.35; *p* = 0.03). Furthermore, we observed an increase in DNA methylation levels in 30 < IAT < 49 group compared to the IAT > 50 at CpG 5 (30 < IAT < 49: 23.87 ± 1.95; IAT > 50: 14.78 ± 2.21; *p* < 0.0001). Moreover, we also reported a significant increase in DNA methylation at CpG 5 in the IAT > 50 group compared to the IAT < 29 group (IAT > 50: 14.78 ± 2.21; IAT < 29: 10.50 ± 0.55; *p* = 0.042) (Fig. 2b).

**Figure 3:**
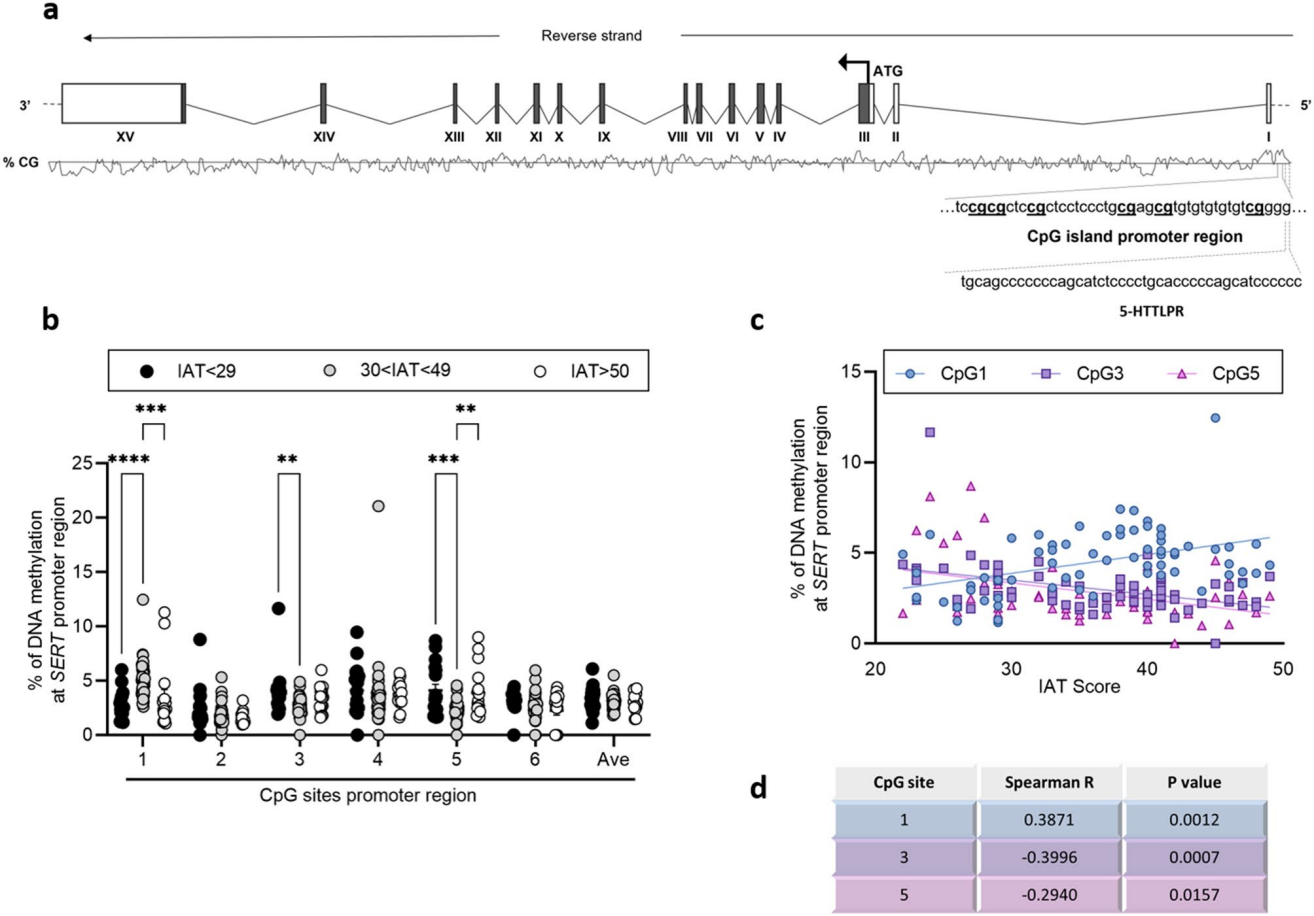
(**a**) Schematic representation of the human *SERT* gene. Boxes represent the exons, with the translated part, beginning on the ATG site, filled in dark grey. In the lower part are reported the CpG island located into the promoter region in which are highlighted the 6 CpG sites under study and the region containing the 5-HTTLPR. (**b**) DNA methylation levels in *SERT* promoter region in saliva samples of young adults with IAT < 29, 30 < IAT < 49 and IAT > 50, represented as scattered dot plots (mean ± SEM, of each group) for the individual CpG sites and the average (Ave) of the 6 CpG sites included in the study. Significant differences are indicated (Bonferroni corrected **p < 0.01, ***p < 0.005, ****p < 0.001). (**c**) Correlation between % of DNA methylation at *SERT* promoter region (Y bar) and IAT score (X bar). Data were compared by Spearman’s rank correlation coefficient; Spearman’s r and *p* values for each CpG site are reported in (**d**).

With respect to *SERT* gene (schematic representation Fig. 3a), we observed an increase in DNA methylation at CpG 1 in students reporting 30 < IAT < 49 score compared to those with IAT < 29 (30 < IAT < 49: 4.93 ± 0.16; IAT < 29: 2.82 ± 0.31; *p* < 0.0001) and IAT > 50 (30 < IAT < 49: 4.93 ± 0.16; IAT > 50: 3.43 ± 0.75; *p* = 0.002). Instead, a decrease in DNA methylation was observed in the group of young adults with 30 < IAT < 49 compared to the group with lower IAT score at CpG 3 (30 < IAT < 49: 2.67 ± 0.11; IAT < 29: 4 ± 0.49; *p* = 0.005) and at CpG 5 (30 < IAT < 49: 2.35 ± 0.11; IAT < 29: 4.10 ± 0.56; *p* = 0.0002). Furthermore, we showed a decrease in DNA methylation levels in 30 < IAT < 49 group compared to the IAT > 50 at CpG 5 (30 < IAT < 49: 2.35 ± 0.11; IAT > 50: 3.91 ± 0.57; *p* = 0.0015) (Fig. 3b).

Considering men and women individually, we observed that there were no substantial differences in DNA methylation levels between males and females, whereas the number of males was quite low (see Supplementary Fig. 1). Taking into account the differences in DNA methylation levels at the three gene regions between the three subgroups, we did not observe interactions with the gender of the subjects, except for *SERT* CpG 1 (F (2, 77) = 4.606, *p* = 0.0129), and *DAT1* CpG 3 (F (2, 78) = 4.405, *p* = 0.0154) and 7 (F (2, 78) = 4.777, *p* = 0.0110) (Supplementary Fig. 2). Moreover, there were no differences in DNA methylation levels considering the age of the subjects involved in the study (data not shown), while a significant negative correlation was observed between subjects’ age and *OXTR* DNA methylation levels at CpG 2 (Spearman r = − 0.2788; *p* = 0.0107) and CpG 3 (Spearman r = -−0.2764; *p* = 0.0114) (Supplementary Table 1).

### Correlation analysis

Spearman’s correlations analysis revealed selective and significative direct correlation between IAT score and *OXTR* DNA methylation levels at CpG 2 (Spearman r = 0.443; *p* = 0.0002), CpG 3 (Spearman r = 0.584; *p* = 0.0002), CpG 4 (Spearman r = 0.272; *p* = 0.024), and with the average of the 4 CpG sites (Spearman r = 0.346; *p* = 0.038), in subjects with IAT < 50 (Fig. 1c). In addition to *OXTR*, in the same group, a significant direct correlation was observed with *DAT1* DNA methylation levels at CpG 1 (Spearman r = 0.484; *p* < 0.0001), CpG 5 (Spearman r = 0.312; *p* = 0.009), and with the average of the 6 CpG sites (Spearman r = 0.473; *p* < 0.0001) (Fig. 2c). Finally, we observed a significant direct correlation between *SERT* DNA methylation levels at CpG 1 and IAT score, again considering only subjects with IAT < 50 (Spearman r = 0.387; *p* = 0.0012). However, Spearman’s correlation analysis showed a significant negative correlation between IAT score and *SERT* DNA methylation levels at CpG 2 (Spearman r = − 0.292; *p* = 0.016), CpG 3 (Spearman r = − 0.399; *p* = 0.0007), CpG 5 (Spearman r = − 0.294; *p* = 0.015), and with the average of the 6 CpG sites (Spearman r = − 0.258; *p* = 0.033) (Fig. 3c).

**Figure 4:**
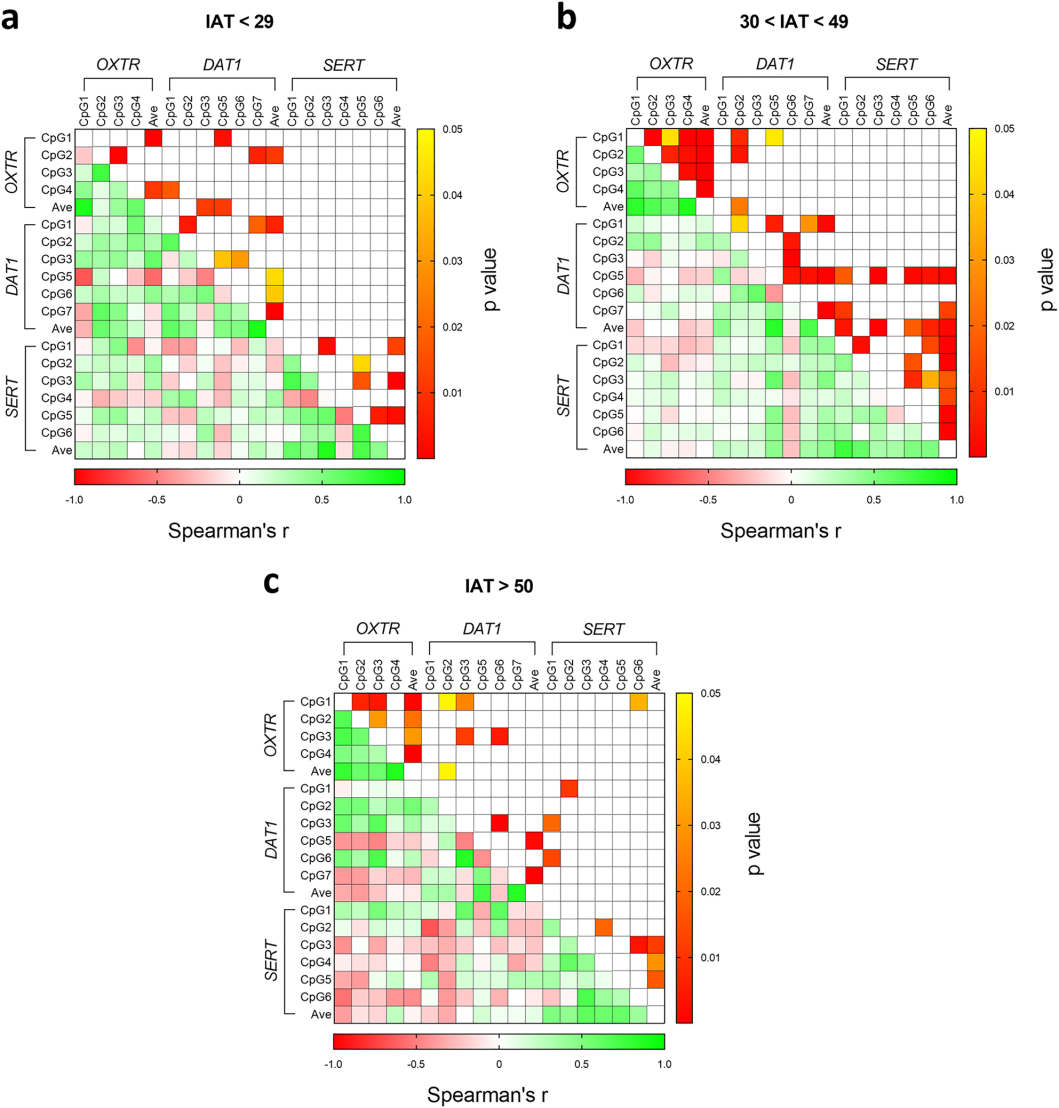
Heat maps representing the correlation analysis between the subjects’ IAT score and DNA methylation levels in each CpG site of the three genes under study. Cells filled in green to red gradient of the heat maps (lower part) represent Spearman’s r; cells filled in yellow to red gradient (upper part) represent *p* values (empty cells stand for *p* values greater than 0.05). Subjects under study are divided considering their IAT score less than 29 (**a**), between 30 and 49 (**b**), or greater than 50 (**c**).

**Figure 5:**
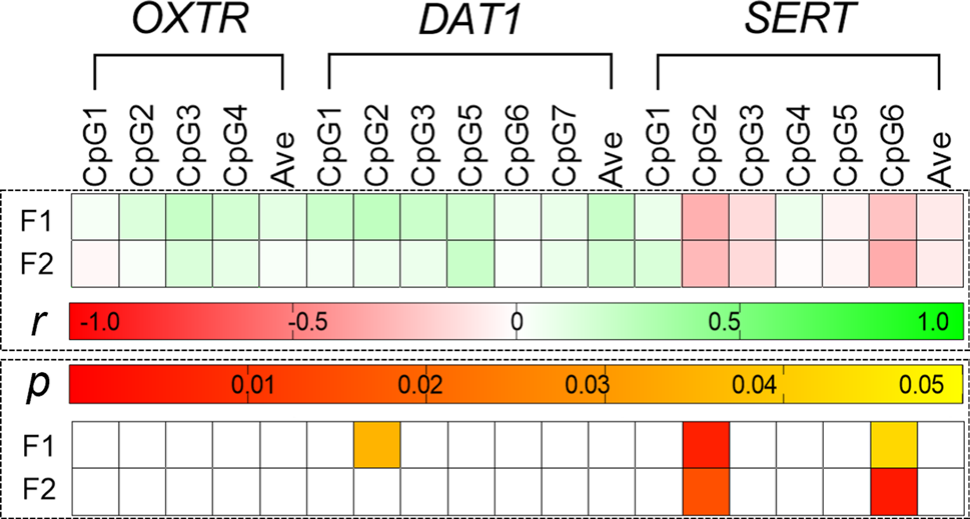
Heat maps representing the correlation analysis between the subjects’ IAT score considering the single psychometric factor (F1 and F2) and DNA methylation levels at each CpG site of the three genes under study. Cells filled in green to red gradient of the heat maps (upper part) represent Spearman’s r; cells filled in yellow to red gradient (lower part) represent *p* values (empty cells stand for *p* values greater than 0.05).

To summarise all correlation data, we reported heatmaps showing correlation between DNA methylation levels at all the CpG sites under study in the different genes (Fig. 4). As it can be seen in the figures, the correlation matrix for all the CpG sites under study were stronger when considering the mild group (30 < IAT < 49) (Fig. 4b) with respect to the other two subgroups (IAT < 29 and IAT > 50) (Fig. 4a and 4c, respectively). More in detail, considering *DAT1* CpG sites also segregated by genotype (Supplementary Fig. 5), we observed a statistically significant negative correlation between CpG 3 and 5 in IAT < 29 subjects (mainly driven by 10/10 subjects), that gets stronger when looking at 30 < IAT < 49 subjects of 10/10 genotype only (Supplementary Fig. 5 panel e). On the other hand, still within 10/10 subjects, the negative significant correlation between *DAT1* CpG 5 and 6 was observed in 30 < IAT < 49 subjects and was not present in the IAT < 29 subgroup. Moreover, considering again *DAT1* CpG 5 and 6, it can be observed in IAT < 29 subjects that DNA methylation at CpG 5 was inversely correlated to *SERT* CpG sites, whereas CpG6 was directly correlated to *SERT* CpG sites, even if not significantly (Fig. 4a). When considering 30 < IAT < 49 subjects, we observed the exactly opposite tendency: DNA methylation at *DAT1* CpG 5 is directly correlated with DNA methylation at all *SERT* CpG sites, which serves an opposite profile compared to the inverse correlation that *SERT* CpG sites have with DNA methylation at *DAT1* CpG 6. All these correlations for *DAT1* CpG 5 were significant considering *SERT* CpG 1, CpG 3, CpG 5, CpG 6, as well as the average of all *SERT* CpG sites (Fig. 4b). Similar correlation pathways can be observed considering *OXTR* and *SERT* CpG sites methylation, again with 30 < IAT < 49 subjects showing the strongest correlation compared to the other two groups, besides the correlation between *OXTR* CpG 1 and *SERT* CpG 6 in the IAT > 50 subgroup (Fig. 4c). We also analysed the data considering the two psychometric factors of the IAT test, observing that the most relevant changes occur at *DAT1* CpG 2 and *SERT* CpG 2 and CpG 6 (Fig. 5).

**Figure 6:**
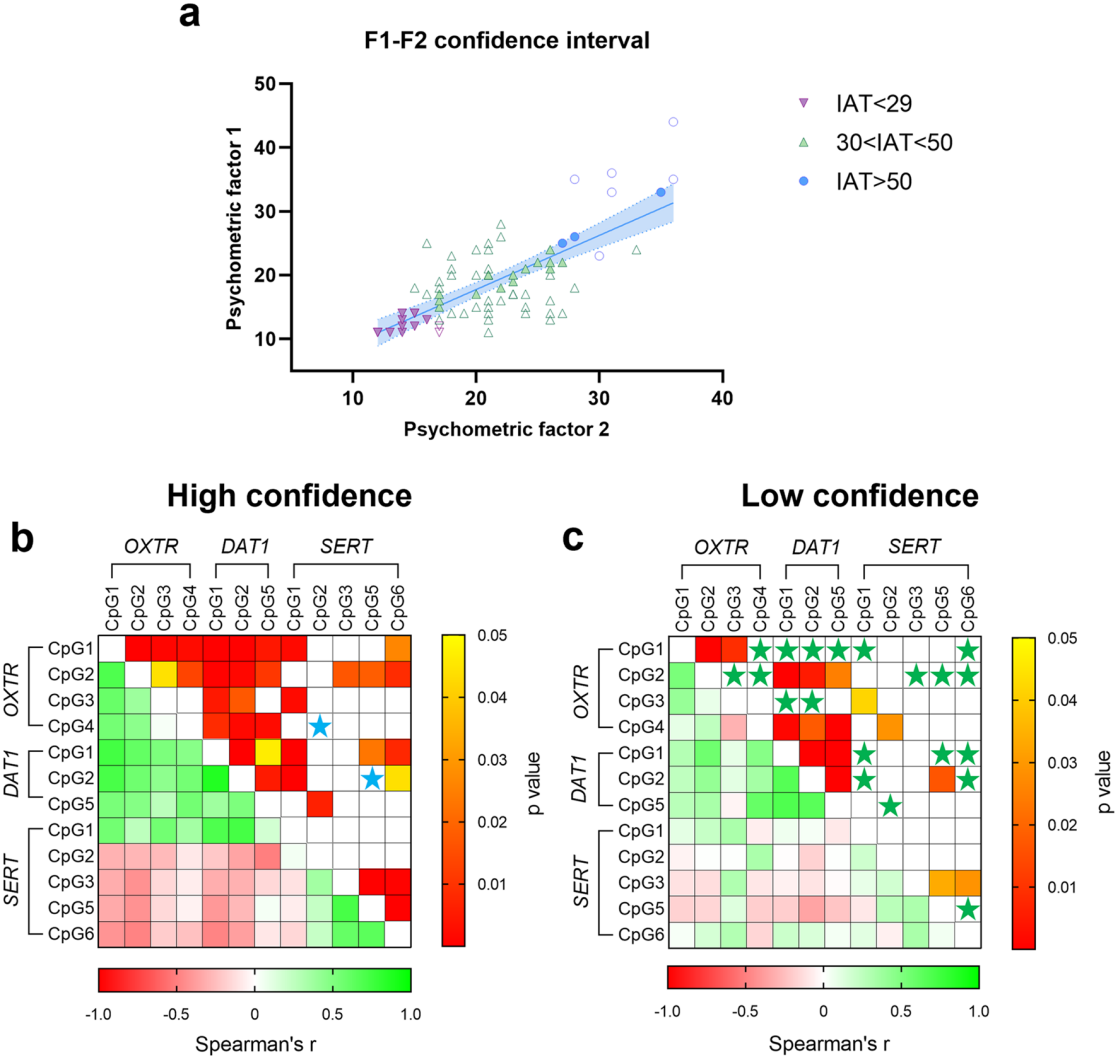
(**a**) Correlation between the two psychometric factors: subjects are divided considering their high (filled symbols inside the 95% confidence interval) or low confidence (empty symbols outside the 95% confidence interval). Heat maps representing the correlation analysis between DNA methylation levels at the CpG sites of the three genes reported to be statistically different between the IAT groups or correlating with F1 and F2 (Fig. 5). Subjects are divided considering their High (**b**) or Low (**c**) F1-F2 confidence interval. Cells filled in green to red gradient of the heat maps (lower part) represent Spearman’s r; cells filled in yellow to red gradient (upper part) represent *p* values (empty cells stand for *p* values greater than 0.05). Blue (**b**) and green (**c**) stars show the missed correlation between two CpG sites comparing the two groups of subjects.

Finally, correlating the F1 and F2 scores, we divided our study samples into two groups: subjects with a high F1-F2 confidence, inside the 95% confidence interval of the linear correlation line, and subjects with a low F1-F2 confidence, outside of the 95% confidence interval (Fig. 6a). Considering those CpG sites with significant differences between IAT sub-groups (Figs. 1, 2, 3, and 5) in those falling inside the F1-F2 95% confidence interval (filled symbols in Fig. 6a) there was a significant correlation between the majority of *OXTR*, *DAT1* and *SERT* CpG sites (Fig. 6b), that was lost in those subjects outside the interval thus with lower F1-F2 correlation (Fig. 6c).

### Genetic analysis

**Figure 7:**
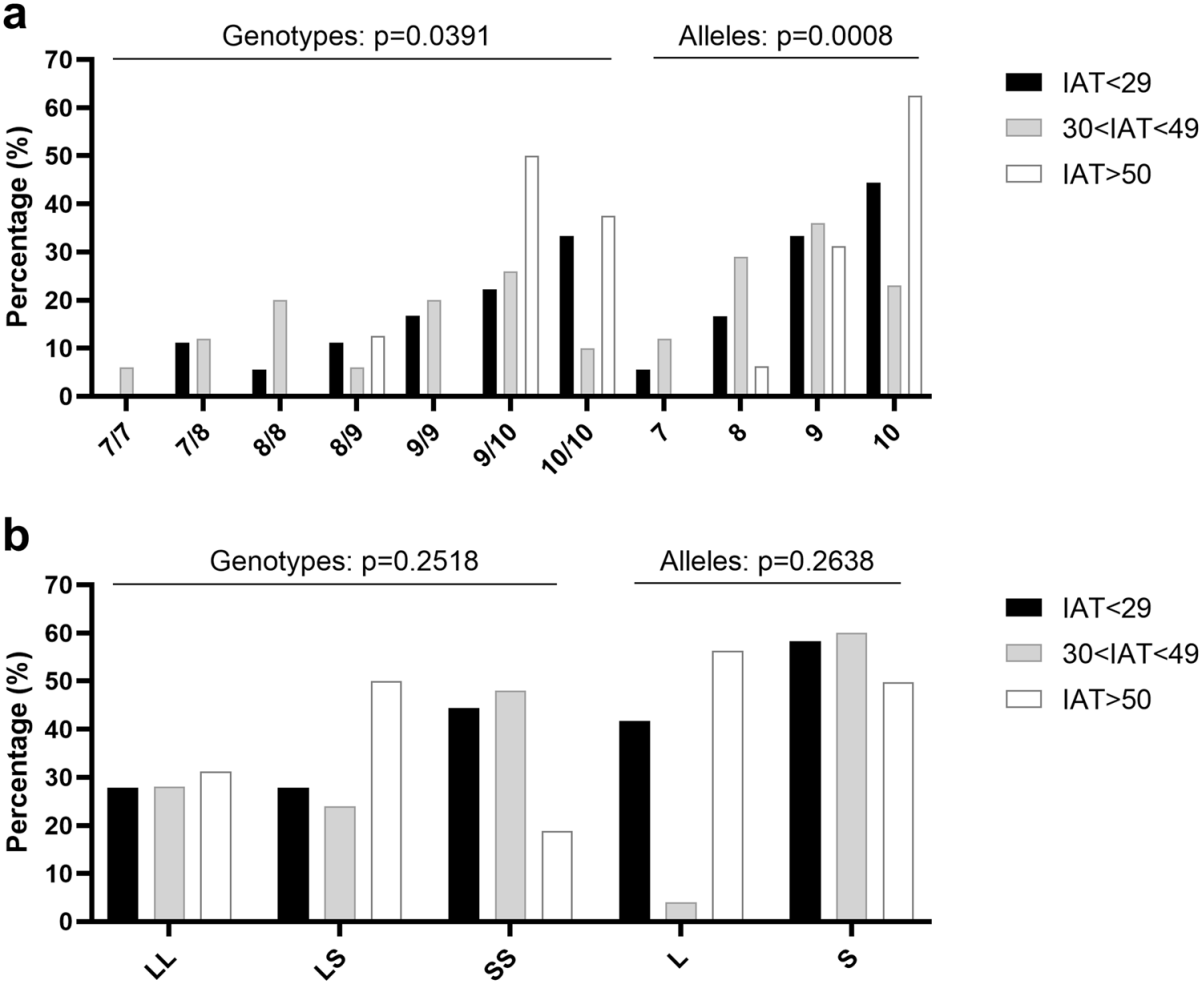
Genotypes and alleles distribution of *DAT1* VNTR (**a**) and *SERT* 5-HTTLPR (**b**) between the groups. Box plots represent the percentage of subjects from each group with the specific genotype and allele. Data were analysed with the Chi-square test. *P* values are reported above.

We finally analysed the repetition frequency of the 40bp-VNTR located into the 3’UTR of *DAT1* gene and the chi-square test revealed a significant difference for genotype (χ2 21.86; *p* = 0.0391) and allele distribution (χ2 22.99; *p* = 0.0008) as a function of the subjects’ IAT score classification (Fig. 7a and Table 2). We did not observe significant differences between the groups in either genotype (χ2 5.37; *p* = 0.2518) or allelic distribution (χ2 2.67; *p* = 0.2638) for the 5-HTTLPR polymorphism (Fig. 7b and Table 2).

**Table 2:** Genotypes and alleles distribution of *DAT1* VNTR and *SERT* 5-HTTLPR between the groups. Data are reported both in number and percentage for each genotype and allele. Data were analysed with the Chi-square test. *P* values are reported on the right.

Internet addiction test score (IAT)					
Genotype/Allele	IAT < 29 (18)	30 < IAT < 49 (50)	IAT > 50 (16)	*p*-value	Chi-square, df
*DAT1* VNTR genotypes/alleles (n, %)					
7/7	0 (0)	3 (6)	0 (0)	0.0391	21.86, 12
7/8	2 (11.1)	6 (12)	0 (0)		
8/8	1 (5.56)	10 (20)	0 (0)		
8/9	2 (11.1)	3 (6)	2 (12.5)		
9/9	3 (16.67)	10 (20)	0 (0)		
9/10	4 (22.24)	13 (26)	8 (50)		
10/10	6 (33.33)	5 (10)	6 (37.5)		
7	2 (5.56)	12 (12)	0 (0)	0.0008	22.99, 6
8	6 (16.66)	29 (29)	2 (6.25)		
9	12 (33.34)	36 (36)	10 (31.25)		
10	16 (44.44)	23 (23)	20 (62.5)		
*SERT* 5-HTTLPR genotypes/alleles (n, %)					
LL	5 (27.8)	14 (28)	5 (31.2)	0.2518	5.365, 4
LS	5 (27.8)	12 (24)	8 (50)		
SS	8 (44.4)	24 (48)	3 (18.8)		
L	15 (41.7)	40 (40)	18 (56.25)	0.2638	2.665, 2
S	21 (58.3)	60 (60)	14 (43.75)		

No differences were observed looking at *SERT* DNA methylation levels considering 5-HTTLPR genotype (Supplementary Fig. 3a), not even considering IAT score and 5-HTTLPR genotype at the CpG sites found to be differentially methylated (CpG1, CpG3, and CpG5) when subjects were compared based on the IAT score (Supplementary Fig. 3b). Looking at *DAT1* 5′UTR DNA methylation levels considering *DAT1* VNTR genotype, an increase in DNA methylation levels at CpG5 was observed in subjects with 7/7 genotype compared to 8/9, 9/9, 9/10, and 10/10, as well as higher levels at the same CpG site in subjects with 8/8 genotype respect to those with 8/9, 9/9, 9/10, and 10/10 (Supplementary Fig. 4a). No differences in *DAT1* 5’UTR DNA methylation levels were instead observed between subjects with the 9/9, 9/10, and 10/10 genotypes, with the 9 and 10 repeat polymorphisms being the most common alleles present in the general population. Moreover, looking at subjects’ distribution based on the IAT score, the moderate IA subjects are those with the highest DNA methylation levels, regardless of genotype (Supplementary Fig. 4b).

## Discussion

In this study we observed that young adult university students, stratified on different scores on the IAT, showed different epigenetic regulation of key genes (*OXTR*, *DAT1*, and *SERT*) that might potentially suggest a predisposition to develop an addictive behaviour. The most relevant changes in the epigenetic mark we here considered, DNA methylation, occurred in those subjects with mild level (30 < IAT < 49) of IA when compared to those with a normal level of Internet usage (IAT < 29) and to those that can be considered already addicted even if moderately (50 < IAT < 80). In our study sample no subjects reported severe IA.

These data might appear paradoxical; however, we believe that it is of relevance the epigenetic timing and the potential of DNA methylation changes as early biomarkers to be taken into account in order to avoid a potential addiction. It has been in fact already reported that epigenetic markers detectable at early stages can be transient and not measurable later in disease progression or life^47,48^.

Our analysis also shed the light on significant correlations between the alterations observed in the epigenetic mark for all the three genes. This was still evident when considering two psychometric factors of the IAT test and, likely, in those subjects with mild level of IA but not in those with moderate level as well as in subjects with a normal use of the Internet.

Specifically, we reported alterations in *OXTR* DNA methylation at exon III already found to be modulated based on different social environments^28,29,49^. It has been reported that Trier social stress test, an ecologically valid stressor based on the stress induced by public speaking, induces a rapid increase in *OXTR* DNA methylation levels within exon III, that is restored to normal levels just 90 min after performing the test^29^. The same mechanism regulating *OXTR* expression has been linked also to adverse early life experiences, with higher levels of DNA methylation for some of the CpG sites here investigated observed in adults reporting during childhood low maternal care compared to those reporting high maternal care^50^. Altered DNA methylation levels in the same region have been also previously associated with central nervous system disorders such as obsessive–compulsive disorder^30,51,52^, social anxiety disorder^53^, autism^54^ and depression^55^.

The same pattern of changes in DNA methylation was observed at *DAT1* gene promoter, alterations already reported in relation to mental health problems and addiction^56,57^. In contrast to *OXTR* gene in which all the CpG sites under study resulted to be more methylated in the 30 < IAT < 49 group, only two *DAT1* CpG sites, numbered 1 and 5, resulted to have higher level of DNA methylation in the same group when compared to the other two. Our molecular data are consistent with previous works focusing on other forms of addictions, such as alcohol dependence and in particular in subjects under craving^57^ and Internet Gaming Disorder^58^. This increase in *DAT1* DNA methylation should evoke a reduction in its expression and thus an increase in DA levels in mild Internet users, consistently with that already observed in the striatum of subjects with IA disorder^15^. To further investigate the role of *DAT1* gene regulation in IA, subjects were genotyped for *DAT1* VNTR, a polymorphism already associated with neuropsychiatric disorders^59,60^. Between 3 and 11 copies of the 40 bp VNTR have been identified in normal populations, and the 9 and 10 repeats alleles are the most frequent^61,62^, also in our study sample. An association between the IAT scores and the 9/10 and 10/10 genotypes as well as the 10 allele emerged, but there were no differences among these genotypes in gene DNA methylation levels. In a previous work from our group, we reported that the 10 repeats allele confers resistance to treatment in patients with Attention Deficit Hyperactivity Disorder and it was also correlated with higher DNA methylation in the very same CpG1 in the *DAT1* gene promoter region^56^. Overall, our results on *DAT1* gene regulation support previous findings showing an association between a dysfunction of the dopaminergic system and subjects with excessive use of the Internet^15,17^.

Many addictive drugs act on DAT, thereby increasing extracellular DA concentration in key brain regions, however many can also modulate 5HTT, and thus increasing extracellular serotonin concentrations. The abuse liability of a drug (i.e., the tendency of a drug to be used in non-medical situations, even sporadically, due to underlying psychoactive effects it produces) has been associated with a different affinity for these two transporters. In detail, those having higher affinity for DAT were associated with a higher addictiveness of the drug, whereas other drugs which are more akin to 5HTT were less addictive. Thus, we also monitored DNA methylation at *SERT* gene, coding for 5HTT, which exhibited a high degree of sequence similarity with the *DAT1* but with different expression patterns suggesting differences in their regulation^63^. Interestingly, beside the increase at CpG site number 1 in the 30 < IAT < 49 group when compared with the other two groups, we also observed a significant reduction in *SERT* DNA methylation levels at two CpG sites (3 and 5), thus in the opposite direction of *DAT1* methylation. It might be suggested that the possible increase in DA levels is counterbalanced by an increase in 5-HT levels in those subjects with higher IAT scores. Interactions between serotonergic and dopaminergic systems have been reported^64^ and a functional modulation of 5-HT over DA activities in the neural network observed^65^. For example, deficient serotonergic function may result in hyperactivity of the DA system, promoting impulsive behaviours’^66,67^. On the other hand, we also performed bioinformatic analysis using PROMO^68,69^ in order to identify transcription factors (TFs) predicted to bind *SERT* sequence, and we observed that FACB and ZF5 selectively bind CpG 1, FACB, ZF5 and p300 bind CpG 3, Msx-1, HOXA3, ZF5, and HES-1 bind CpG 5. Among these, it is interesting to note that just for p300, selective for CpG 3, and HES-1, selective for CpG 5, the two CpG sites where DNA methylation resulted to be reduced in the mild-IA group, their recruitment has been already associated to gene activation^70,71^. Moreover, other human studies showed that epigenetic regulation of *SERT* promoter region is associated with behavioural differences in response to environmental stressors^72,73^. We also analysed *SERT* repeat length polymorphism (5*-*HTTLPR) consisting of a variation of the repetitive sequence containing GC-rich, 20–23-bp-long repeat elements. A deletion/insertion in the 5-HTTLPR was reported to form the 14 repeats as short (S, low expressing) and the 16 repeats as long (L, high expressing) alleles. Lee and Colleagues ^22^ showed that the S allele was more frequent in excessive Internet users, while, conversely, we did not observe significant association between IA and 5-HTTLPR polymorphism.

When considering the possible association occurring among the epigenetic mark at the level of the three genes under study and the IAT scores analysing the different population subgroups, we can still observe that most of the significant changes occur in the mild group. Of particular interest appears the inverse correlation between *DAT1* CpG 3 and CpG 5 in the group with lower IAT scores (IAT < 29): within 9/x subjects, it is no longer evident in the mild group (30 < IAT < 49). Here, within 10/10 subjects, an inverse correlation occurs between CpG3 and CpG5 as well as between CpG 5 and CpG 6. A previous study by some authors involved in the present work^17^ reported an anti-correlation between these *DAT1* CpGs (3 and 5) in control individuals (youth with lower IAT and BIS [Barrat Impulsivity Scale] scores). When we further stratify our data considering the subjects’ genotype for *DAT1* VNTR, we notice an anti-correlation between CpG3 and CpG5 as well as between CpG 5 and CpG 6 in 30 < IAT < 49 individuals, but only for those carrying the 10/10 genotype (Supplementary Fig. 5). In addition, we also report that DNA methylation at *DAT1* CpG 5 and CpG 6 are directly correlated with that at *SERT* CpGs in the IAT < 29 group. Once again, the direction of these correlations resulted in the opposite direction in the mild problematic group (30 < IAT < 49).

Likewise, at *OXTR* gene we have observed an intra-motif link. In particular, all *OXTR* CpG sites show a strong positive and significant correlation with each other in the mild group, whereas the same mechanism is not visible in the other two groups (control and moderate internet users).

When we took into account the two psychometric factors of the IAT, we showed that just the first one, representing the preoccupations linked to the social consequences of Internet use, was significantly correlated to DNA methylation levels of *DAT1* CpG 2. Moreover, both psychometric factors were inversely correlated to DNA methylation at *SERT* CpG 2 and CpG 6.

Finally, considering DNA methylation correlations among the different genes under study in those subjects with lower F1-F2 correlation (Low confidence) compared with those with higher F1-F2 correlation (High confidence), we observe that significant changes were evident just in the latter.

Our data overall attempt to suggest that changes in genes DNA methylation might be biological markers associated with altered behaviour (i.e., Internet use) that might eventually predispose to a full-blown manifestation of IA. The changes occurring in the 30 < IAT < 49 group appear to be of particular interest since these might suggest that early alterations in genes regulation might evoke an altered transitory modulation of their expression. However, if a potential addictive behaviour occurs, as observed considering the group of subjects with the highest IAT score, these epigenetic changes are no more evident, drawing attention to the potential predictive nature of these marks. We thus highlight the significance of epigenetic timing since, as mentioned above, alterations in epigenetic markers detectable at early stages can be transient and not measurable later in disease progression or life^48,74^. These timing effects could also explain some inconsistent findings reported in the literature^75^. We thus believe that the identification of early epigenetic biomarkers would be of relevance in order to predict disease trajectories and eventually choose a suitable therapy. Considering our study population, several social and emotional environmental factors, such as academic performance, social isolation, or public speaking, should be taken into account as possible triggers of the epigenetic alterations. The role of gene VNTRs seemed less relevant, allowing us to suggest that the altered behaviour in our study samples might be driven by environmental triggers, rather than by a genetic background.

Finally, the occurrence of the epigenetic mark in function of the association between the two psychometric factors highlights the possibility of detecting sub-phenotypes of this condition. In fact, subjects with the same IAT score might have different low or high correlation between psychometric factors. This should be considered in order to predict not only the risk to eventually develop a disorder (i.e. IA), but also a potential altered behaviour that might be relevant for a healthy condition.

### Study limitations

This study has some potential limitations, like the relatively small sample size (N = 84) even if the achieved power for the DNA methylation study for each path was ≥ 0.8. All samples were collected in a single time-point, and it would be of clear relevance to run a longitudinal study, with multiple time points across days or even within a day, to better understand how the environmental input can modulate the epigenetic marks. Another limitation is represented by the gender distribution of the participants: the majority of the participants are women (N = 64), thus not allowing a proper comparison between sexes. Finally, for selected genes with a potential role in the disorder, we investigated only DNA methylation levels, without looking at their relative expression: this is a technical limitation due to the low-quality degree of total RNA obtainable from saliva. Joint measures of DNA methylation and mRNA expression could allow to better investigate the modulation of key genes as a potential marker for IA developmental trajectories.

Further studies are thus needed to replicate and expand what observed here, including an increased number of participants along with the analysis of other possible key genes. Moreover, it is also needed to address whether an altered DNA methylation status might become fixed or respond to new and positive environmental stimuli over time: this could be of relevance in young adults since, as many studies reported, critical parts of the brain involved in decision-making continue to develop until the early 20 s. The potential reversibility of epigenetic modulation due to changes over time in environmental stimuli might represent a promising therapeutic opportunity.

### Supplementary Information

Supplementary Figure 1.

Supplementary Figure 2.

Supplementary Figure 3.

Supplementary Figure 4.

Supplementary Figure 5.

Supplementary Legends.

Supplementary Table 1.
