# Supplementary figures and images for: Mild internet use is associated with epigenetic alterations of key neurotransmission genes in salivary DNA of young university students
Source: Sci Rep. 2023 Dec 14;13:22192. doi: 10.1038/s41598-023-49492-5 (PMC10719329; doi:10.1038/s41598-023-49492-5)

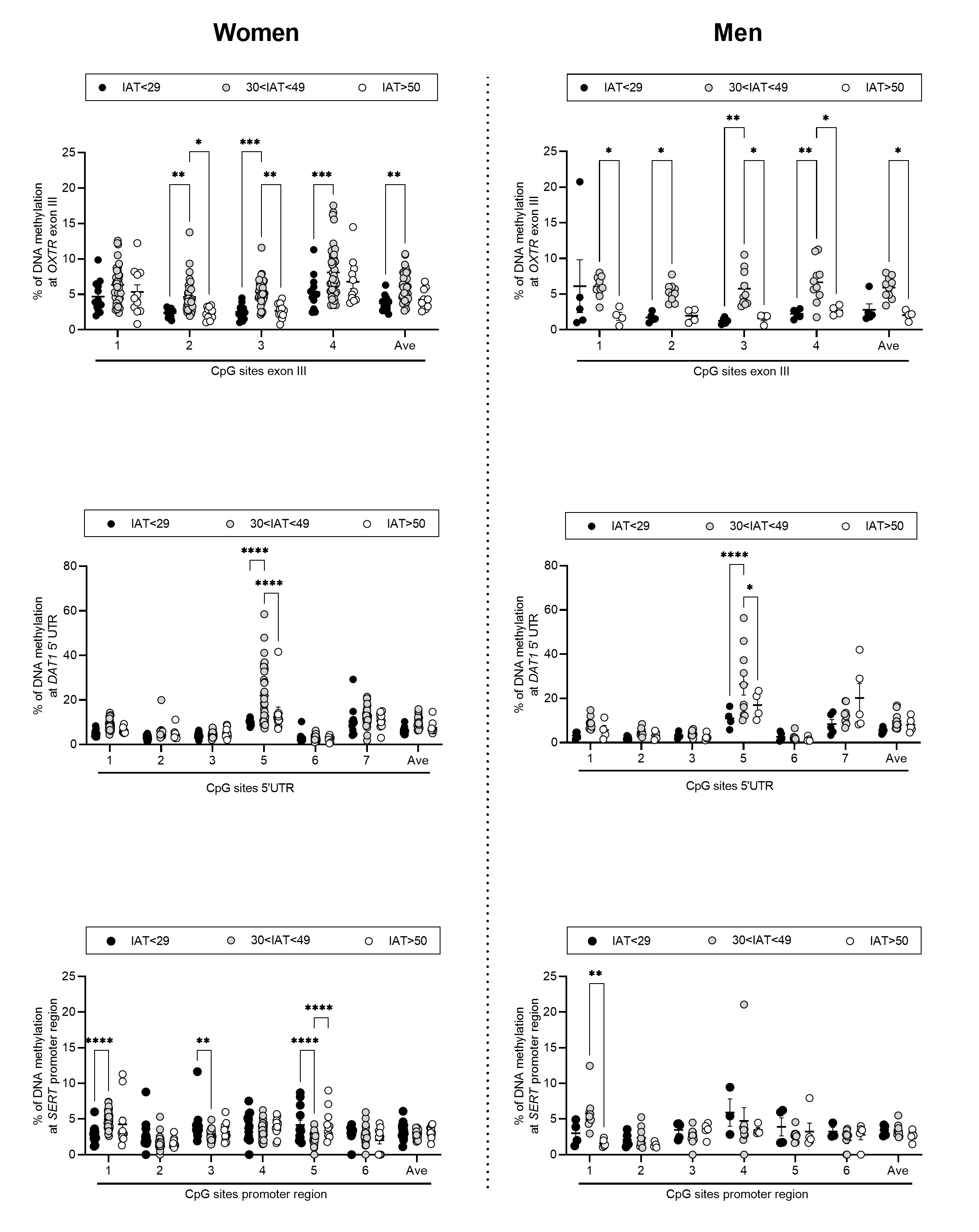

Supplement: Supplementary file 1 — Supplementary Figure 1. [file 41598_2023_49492_MOESM1_ESM.tif]

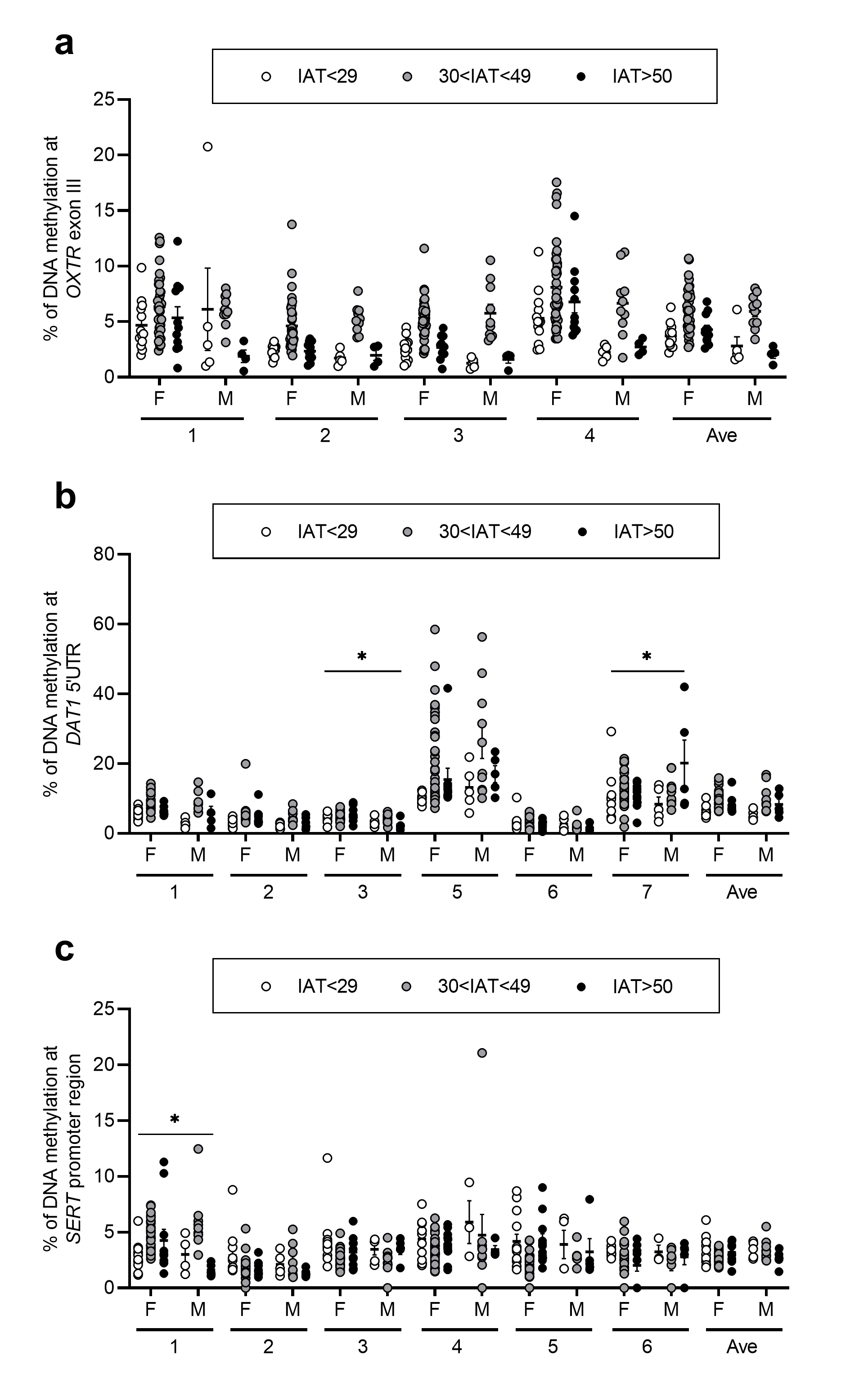

Supplement: Supplementary file 2 — Supplementary Figure 2. [file 41598_2023_49492_MOESM2_ESM.tif]

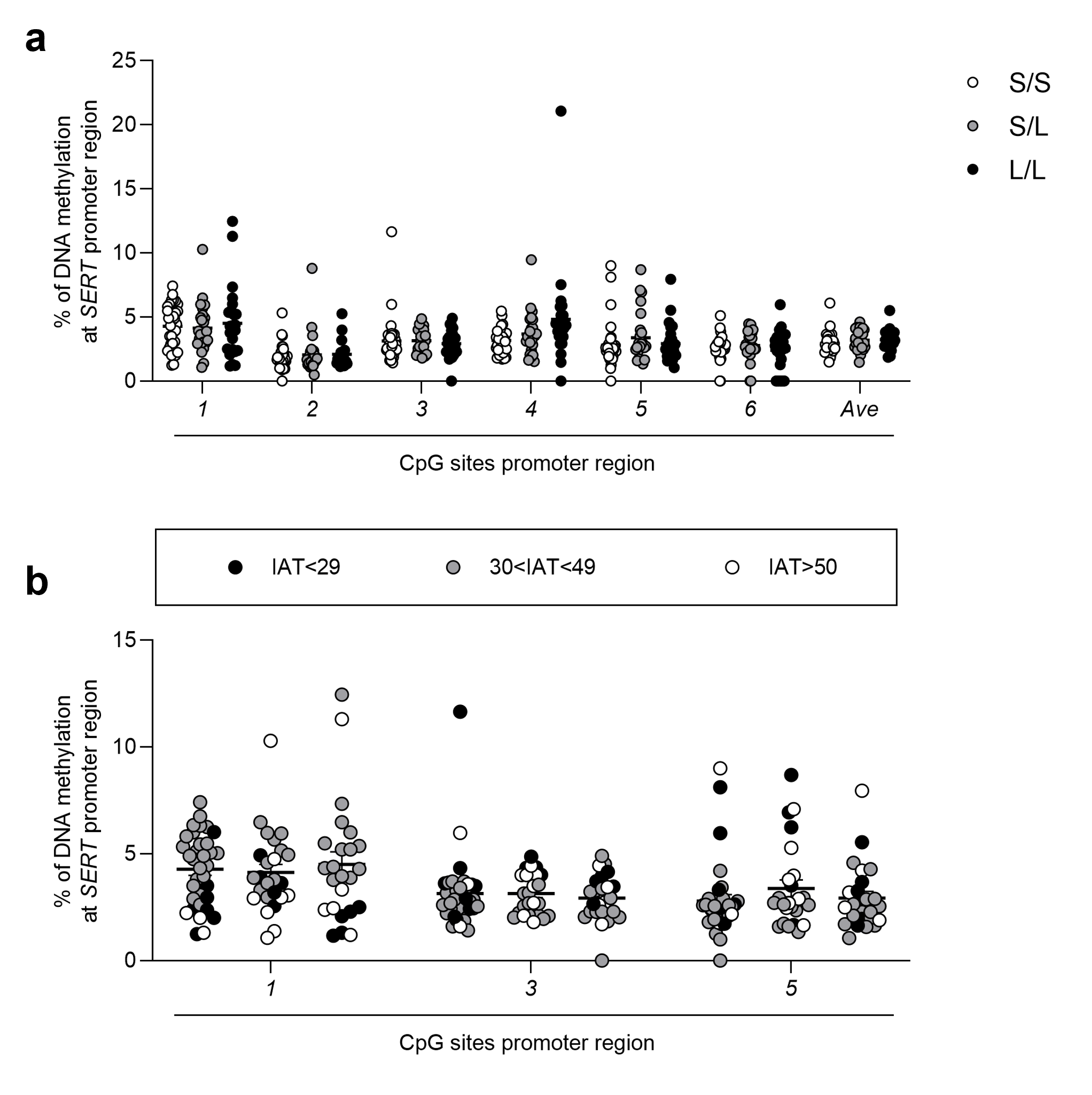

Supplement: Supplementary file 3 — Supplementary Figure 3. [file 41598_2023_49492_MOESM3_ESM.tif]

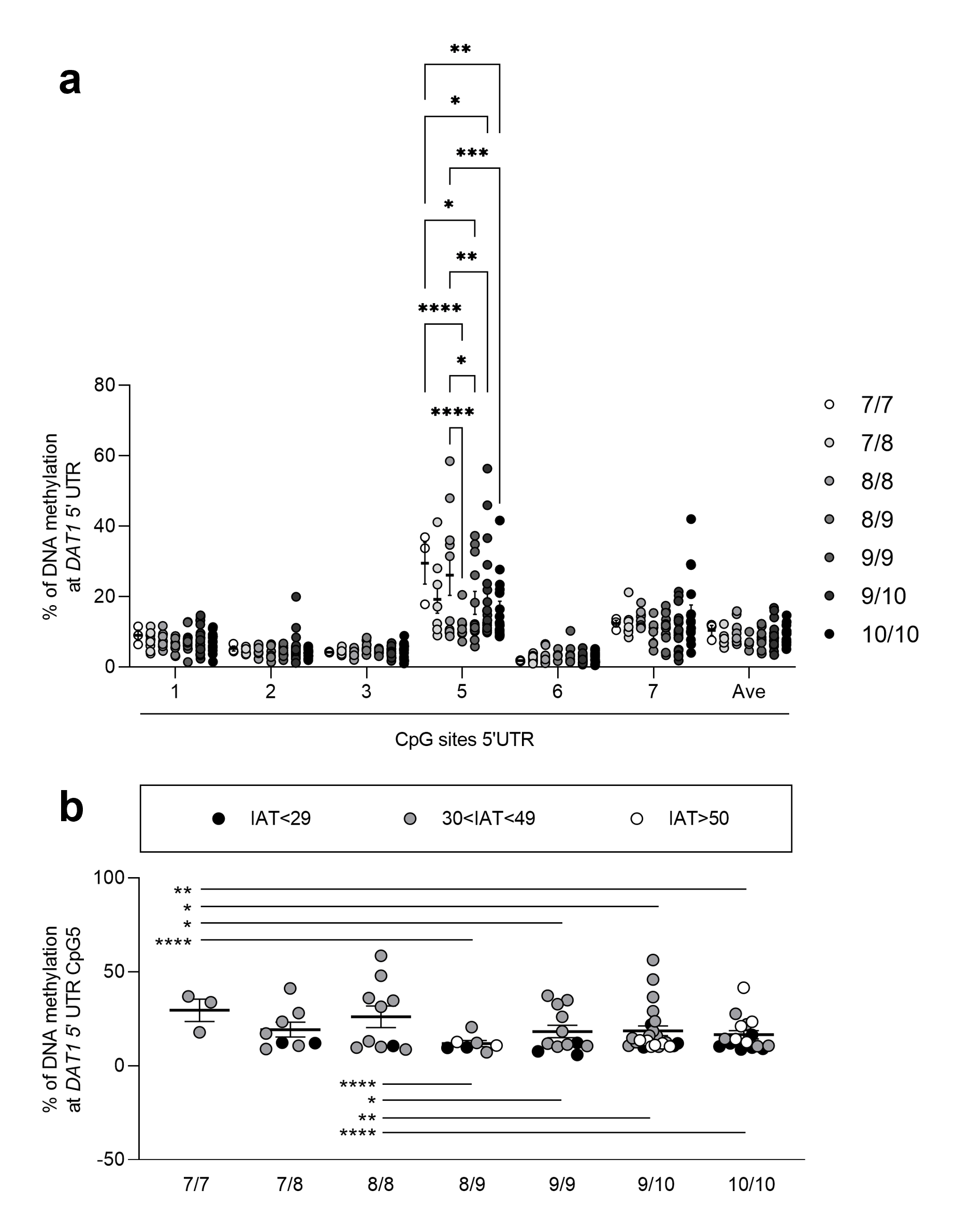

Supplement: Supplementary file 4 — Supplementary Figure 4. [file 41598_2023_49492_MOESM4_ESM.tif]

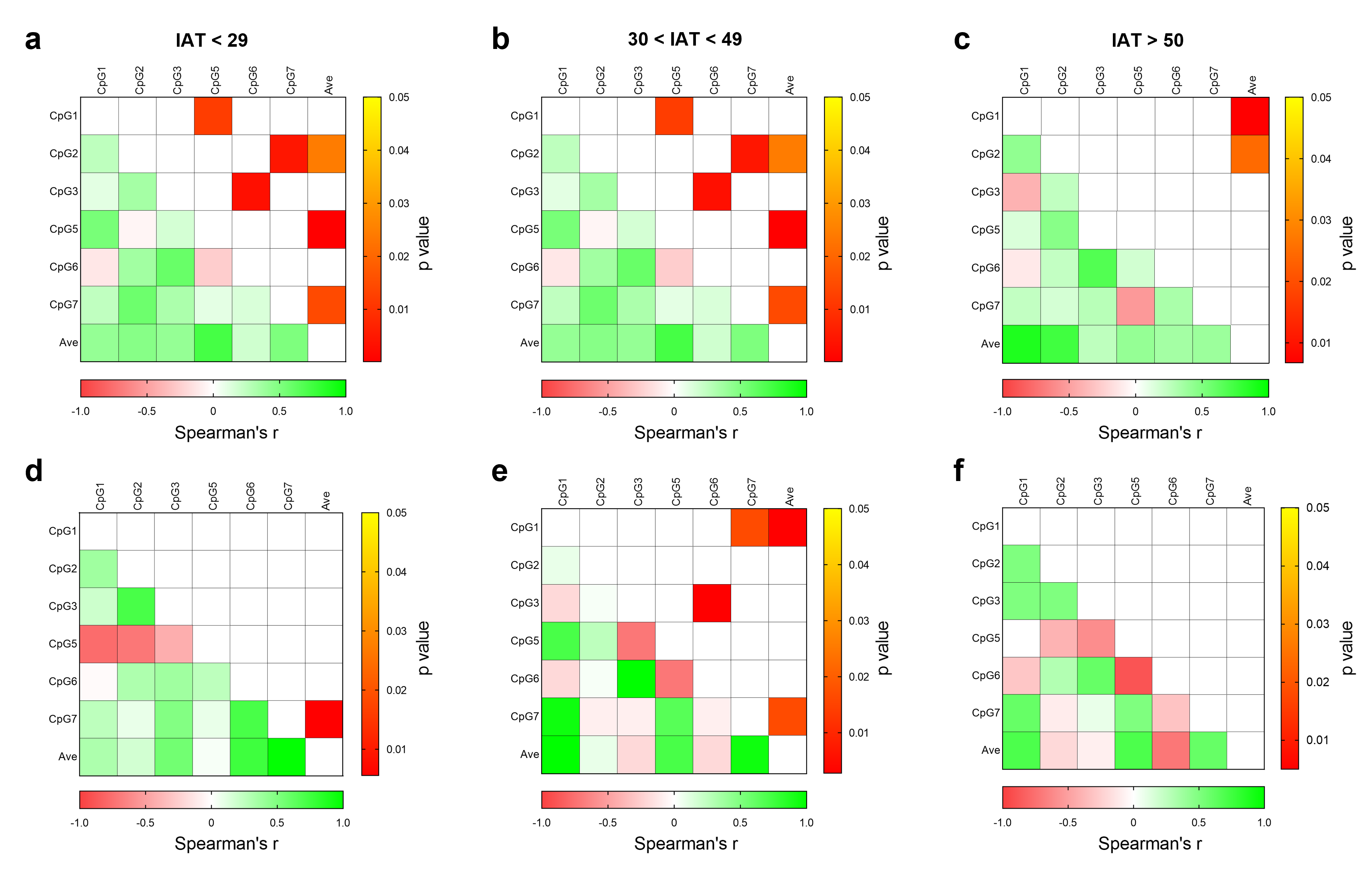

Supplement: Supplementary file 5 — Supplementary Figure 5. [file 41598_2023_49492_MOESM5_ESM.tif]
