# Supplementary material for: Mild internet use is associated with epigenetic alterations of key neurotransmission genes in salivary DNA of young university students
Source: Sci Rep. 2023 Dec 14;13:22192. doi: 10.1038/s41598-023-49492-5 (PMC10719329; doi:10.1038/s41598-023-49492-5)
Supplement: Supplementary file 6 — Supplementary Legends. [file 41598_2023_49492_MOESM6_ESM.docx]

**Supplementary Figure 1.** DNA methylation levels at *OXTR* exon III, *DAT1* 5’UTR, and *SERT* promoter region in saliva samples of women (left) and men (right) young adults with IAT <29, 30<IAT<49 and IAT>50, represented as scattered dot plots (mean ± SEM, of each group) for the individual CpG sites and the average (Ave) of the CpG sites under study. Significant differences are indicated (Bonferroni corrected *p < 0.05, **p < 0.01, ***p < 0.005, ****p < 0.001).

**Supplementary Figure 2.** DNA methylation levels at *OXTR* exon III (**a**), *DAT1* 5’UTR (**b**), and *SERT* promoter region (**c**) in saliva samples of young adults with IAT <29, 30<IAT<49 and IAT>50, divided for gender and represented as scattered dot plots (mean ± SEM, of each group) for the individual CpG sites and the average (Ave) of the CpG sites under study. Significant differences are indicated (Bonferroni corrected *p < 0.05.

**Supplementary Figure 3. a)** DNA methylation levels at *SERT* promoter region in saliva samples of young adults divided for 5-HTTLPR genotype for the individual CpG sites and the average (Ave) of the CpG sites under study. **b**) DNA methylation at *SERT* promoter region CpG sites 1, 3, and 5 in saliva samples of young adults stratified based on their IAT score, represented as scattered dot plots (mean ± SEM, of each group) for the individual CpG sites. The same order of 5-HTTLPR genotype of the picture above is maintained in the picture below (S/S, S/L, and L/L from the left to the right).

**Supplementary Figure 4. a)** DNA methylation levels at *DAT1* 5’UTR in saliva samples of young adults divided for 3’UTR VNTR genotype for the individual CpG sites and the average (Ave) of the CpG sites under study. **b**) DNA methylation at *DAT1* 5’UTR CpG site 5 in saliva samples of young adults stratified based on their IAT score, represented as scattered dot plots (mean ± SEM, of each group) for the individual CpG sites. Significant differences are indicated (Bonferroni corrected *p < 0.05, **p < 0.01, ****p < 0.001).

**Supplementary Figure 5.** Heat maps representing the correlation analysis between *DAT1* DNA methylation levels of CpG sites and the subjects’ IAT score divided for 9/x (**a**, **b**, **c**) and 10/10 genotypes (**d**, **e**, **f**). Cells filled in green to red gradient of the heat maps (lower part) represent Spearman’s r; cells filled in yellow to red gradient (upper part) represent p values (empty cells stand for p values greater than 0.05). Subjects under study are divided considering their IAT score less than 29 **(a, d)**, between 30 and 49 **(b, e)**, or greater than 50 **(c, f)**.

**Supplementary Table 1**. Correlation analysis between subjects’ age and DNA methylation levels. Spearman’s r and p values are indicated; significant values are highlighted in bold (p < 0.05).
