# Supplementary material for: Mild internet use is associated with epigenetic alterations of key neurotransmission genes in salivary DNA of young university students
Source: Sci Rep. 2023 Dec 14;13:22192. doi: 10.1038/s41598-023-49492-5 (PMC10719329; doi:10.1038/s41598-023-49492-5)
Supplement: Supplementary file 7 — Supplementary Table 1. [file 41598_2023_49492_MOESM7_ESM.docx]

| Age x DNA methylation levels | | **Spearman r** | **P value** |
| --- | --- | --- | --- |
| ***OXTR*** | CpG 1 | 0.04761 | 0.6691 |
|  | CpG 2 | -0.2788 | **0.0107** |
|  | CpG 3 | -0.2764 | **0.0114** |
|  | CpG 4 | -0.09037 | 0.4165 |
|  | Average | -0.1043 | 0.3481 |
| ***DAT1*** | CpG 1 | -0.1825 | 0.1007 |
|  | CpG 2 | 0.00776 | 0.9445 |
|  | CpG 3 | 0.008425 | 0.9394 |
|  | CpG 5 | -0.07354 | 0.5088 |
|  | CpG 6 | 0.03185 | 0.7736 |
|  | CpG 7 | -0.09755 | 0.3774 |
|  | Average | -0.0322 | 0.7713 |
| ***SERT*** | CpG 1 | -0.1954 | 0.0766 |
|  | CpG 2 | -0.03974 | 0.7213 |
|  | CpG 3 | -0.01298 | 0.9067 |
|  | CpG 4 | 0.1804 | 0.1116 |
|  | CpG 5 | -0.03629 | 0.7446 |
|  | CpG 6 | -0.008553 | 0.9400 |
|  | Average | 0.02216 | 0.8414 |
